# Fibroblast Growth Factor (FGF) 23 and FGF Receptor 4 promote cardiac metabolic remodeling in chronic kidney disease

**DOI:** 10.21203/rs.3.rs-3705543/v1

**Published:** 2023-12-23

**Authors:** Michaela A. Fuchs, Emily J. Burke, Nejla Latic, Susan Murray, Hanjun Li, Matthew Sparks, Dennis Abraham, Hengtao Zhang, Paul Rosenberg, Sonja Hänzelmann, Fabian Hausmann, Tobias Huber, Reinhold Erben, Kelsey Fisher-Wellman, Nenad Bursac, Myles Wolf, Alexander Grabner

**Affiliations:** 1 Division of Nephrology, Department of Medicine, Duke University School of Medicine, Durham, North Carolina, USA; 2 Department of Biomedical Sciences, University of Veterinary Medicine, Vienna, Austria; 3 Department of Biomedical Engineering, Duke University, Durham, USA; 4 Division of Cardiology, Department of Medicine, Duke University School of Medicine, Durham, North Carolina, USA; 5 Division of Nephrology, Department of Medicine, University Medical Center Hamburg-Eppendorf, Hamburg, Germany; 6 Hamburg Center for Kidney Health (HCKH), University Medical Center Hamburg-Eppendorf, Hamburg, Germany; 7 Ludwig Boltzmann Institute of Osteology, Hanusch Hospital, Vienna, Austria; 8 East Carolina Diabetes and Obesity Institute, Brody School of Medicine, Department of Physiology, East Carolina University, Greenville, North Carolina, USA; 9 UNC Lineberger Comprehensive Cancer Center, University of North Carolina at Chapel Hill School of Medicine, Chapel Hill, NC, USA; 10 Duke Regeneration Center, Duke University, Durham, North Carolina, USA; 11 Duke Clinical Research Institute, Duke University, Durham, North Carolina, USA

**Keywords:** FGF23, FGFR4, CKD, mitochondrial dysfunction, metabolic remodeling, heart failure

## Abstract

Chronic kidney disease (CKD) is a global health epidemic that significantly increases mortality due to cardiovascular disease. Left ventricular hypertrophy (LVH) is an important mechanism of cardiac injury in CKD. High serum levels of fibroblast growth factor (FGF) 23 in patients with CKD may contribute mechanistically to the pathogenesis of LVH by activating FGF receptor (FGFR) 4 signaling in cardiac myocytes. Mitochondrial dysfunction and cardiac metabolic remodeling are early features of cardiac injury that predate development of hypertrophy, but these mechanisms of disease have been insufficiently studied in models of CKD. Wild-type mice with CKD induced by adenine diet developed LVH that was preceded by morphological changes in mitochondrial structure and evidence of cardiac mitochondrial and metabolic dysfunction. In bioengineered cardio-bundles and neonatal rat ventricular myocytes grown in vitro, FGF23-mediated activation of FGFR4 caused a mitochondrial pathology, characterized by increased bioenergetic stress and increased glycolysis, that preceded the development of cellular hypertrophy. The cardiac metabolic changes and associated mitochondrial alterations in mice with CKD were prevented by global or cardiac-specific deletion of FGFR4. These findings indicate that metabolic remodeling and eventually mitochondrial dysfunction are early cardiac complications of CKD that precede structural remodeling of the heart. Mechanistically, FGF23-mediated activation of FGFR4 causes mitochondrial dysfunction, suggesting that early pharmacologic inhibition of FGFR4 might serve as novel therapeutic intervention to prevent development of LVH and heart failure in patients with CKD.

## Graphical Abstract

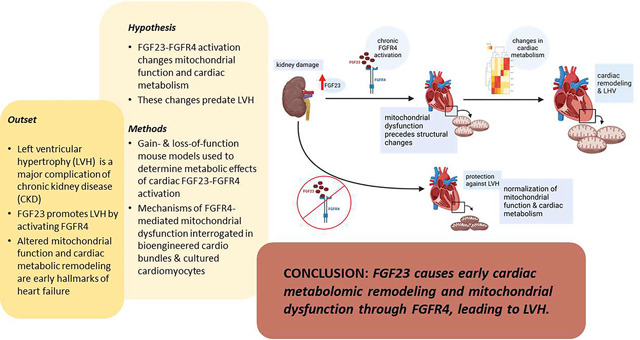

## Introduction

Chronic kidney disease (CKD) is a major health epidemic that affects 37 million adults in the United States and many more worldwide.^1,2^ Patients with CKD face significantly higher risks of cardiovascular disease and subsequent mortality compared to patients without CKD.^3^ Left ventricular hypertrophy (LVH), which affects 50–70% of patients with early and intermediate stages of CKD and up to 90% by the time they reach dialysis, is a major cause of heart failure and in particular heart failure with preserved ejection fraction (HFpEF) in patients with CKD.^4^ Among many factors involved in the pathogenesis of LVH and heart failure, alterations in mineral homeostasis may uniquely contribute in patients with CKD.^5,6^

Fibroblast growth factor (FGF) 23 regulates calcium and phosphate homeostasis.^7^ In CKD, FGF23 levels progressively rise as kidney function declines. Elevated FGF23 initially helps maintain normal serum phosphate in the setting of impaired kidney function,^8^ but higher FGF23 is dose-dependently associated with increased risks of LVH, heart failure and mortality in patients with CKD.^9–11^ Mechanistically, FGF23 directly induces hypertrophic growth of cultured cardiac myocytes and contributes to development of LVH in rodents via activation of FGF receptor (FGFR) 4 and the phospholipase Cγ (PLCγ) – calcineurin – nuclear factor of activated T-cells (NFAT) signaling pathway,^9,12,13^ which is a potent inducer of structural cardiac remodeling.^14^ Human genetic data support the link between FGF23 and development of LVH and heart failure, particularly in patients at risk for developing CKD.^15^

In the pathogenesis of cardiac remodeling that culminates in LVH, mounting evidence suggests that metabolic remodeling and mitochondrial dysfunction precede most, if not all other pathological alterations.^16^ Metabolic inefficiency and loss of coordinated anabolic activity have emerged as proximal causes of cardiac structural remodeling.^17^ Over the past decades, numerous metabolic pathways have been implicated in cardiac hypertrophic growth and heart failure.^18^ However, the role of cardiac metabolism in heart failure specifically in CKD has not been studied extensively, and whether CKD-specific mechanisms, including excess activation of the FGF23–FGFR4 axis, contribute to cardiac metabolic remodeling and mitochondrial dysfunction remains unknown.^19^ In this report, we analyzed cardiac metabolism, mitochondrial composition and function in bioengineered cardio-bundles, cultured cardiomyocytes and multiple rodent models to test the hypotheses that cardiac mitochondrial dysfunction and metabolic remodeling occur in animal models of CKD; that these changes predate the development of structural cardiac remodeling; and that FGF23–FGFR4 signaling are mechanisms of cardiac mitochondrial dysfunction and metabolic remodeling.

## Methods

### Antibodies, recombinant proteins and heparin

Carrier-free recombinant mouse FGF23 (2629FG025/CF) from R&D systems was used at 25 ng/mL and 100 ng/mL. The isoform specific FGFR4 small molecule inhibitor BLU9931 from Selleck Chemicals (Cat. No S7819, TX, USA) was used at 10 ng/mL. Heparin solution (McKesson Corporation, NC, USA) was used at 0.2 USP/mL. Primary antibodies include sarcomeric a-actinin (EA-53, Sigma-Aldrich, USA). Secondary antibodies are Cy3-conjugated goat–anti-mouse (Cat. No. 115165166, Jackson ImmunoResearch Laboratories, PA, USA).

### Isolation and cultivation of neonatal rat ventricular myocytes

Neonatal rat ventricular myocytes were isolated from Sprague-Dawley rat pups (postnatal day 0–3) using a commercially available kit (Cat.No. LK003300,Worthington, NJ, USA) as described previously and in the supplemental methods.^20^

### Fabrication of bio-engineered cardio-bundles

Bioengineered cardio-bundles were generated as described previously and as described in the supplemental methods.^21^ In selected studies, FGF23 (25 ng/mL) and BLU9931 (10 ng/mL) were added to culture medium on day 7 and replenished with each media change during the additional 7 days.

### Measurement of contractile force and action potential propagation

Isometric contractile forces were measured as described previously.^21^ In brief, cardio-bundles were immersed in Tyrode’s solution containing 1.8 mM CaCl2 and connected to a force transducer. Contractions were elicited by electric field stimulus from parallel platinum electrodes. Optical mapping of action potentials was performed as described previously.^22^ Briefly, cardio-bundles were stained with a transmembrane voltage-sensitive dye (di-4-ANEPPS) and paced at different rates by suprathreshold point stimulus to map propagation of action potentials.

### Immunofluorescence and morphometry of cultured myocytes and cardio-bundles

Hypertrophic growth of isolated NRVM was analyzed on laminin-coated glass coverslips after 48 hours of treatment as done previously and as described in the supplemental methods.^12,21,23^

### Live-cell metabolic analysis

Mitochondrial oxygen consumption rate and glycolytic rate were determined in NRVM using the Seahorse XF Mito Stress Test Kit and the Seahorse XF Glycolytic Rate Assay Kit according to manufacturer’s protocols. All live-cell metabolic assays were performed in collaboration with Duke’s Cardiovascular Physiology Core using the metabolic flux analyzer Agilent Seahorse XF96 (Agilent Technologies), see supplemental methods for details and references.

### RNA isolation and quantification

Total RNA was extracted from hearts and cultured cardiomyocytes using a RNeasy Plus Mini Kit (Qiagen) following the manufactures’ instructions. 0.5 – 2 μg of RNA was reverse transcribed to cDNA using Applied Biosystems High-Capacity cDNA Reverse Transcription Kit (cat. No 4368813, ThermoFisher). Quantitative real-time PCR was performed with SSoAdvanced Universal Probe Supermix (Bio-rad) and sequence specific Taqman probes (ThermoFisher) as indicated in Table 1. Samples were run in duplicates on a Quantstudio 3 (Applied Biosystems, ThermoFisher) Real Time detection instrument. Relative gene expression was normalized to expression levels of housekeeping genes β2-microglobulin (for in vitro studies) or 18S rRNA (for in vivo studies). Results were evaluated using the 2^−ΔΔCt^ method and expressed as mean ± standard error of the mean (SEM).

### Mice

Constitutive FGF receptor four null mice (FGFR4^−/−^),^24^ constitutive Col4a3^−/−^ null mice (Alport)^25^ and constitutive FGFR4 knock in mice (FGFR4-Arg385)^26^ were maintained on a C57Bl/6 background. Mice with inducible cardiomyocyte-specific deletion of FGFR4 (α-MHC^MerCreMer^-FGFR4^flox^) were generated by crossing α-MHC^MerCreMer^ mice^27^ with FGFR4 floxed mice.^28^ α-MHC^MerCreMer^-FGFR4^flox^ mice were maintained on a C57Bl/6 background. Cre-recombination was induced by tamoxifen injections (30 mg/kg bodyweight, i.p., every 48 hours for a total of 3 injections). Dietary interventions were started 10 days after the last tamoxifen injection. Male and female mice were used in the distribution indicated in the supplemental methods.

### Adenine model of chronic kidney disease

As done before, chronic kidney disease was induced by feeding 12–16-week-old mice an adenine diet (0.15%, TD.170304 to 0.2% adenine, TD.140290; control diet TD.170303, Envigo, IN, USA)^29^. All mice were put on control diet for one week before study start and then mice were randomized to receive either adenine diet or control diet. After 8, 12 or 16 weeks respectively, mice were sacrificed, plasma collected and hearts were prepared for molecular and histopathologic analyses.

### Non-invasive assessment of kidney function

Glomerular filtration rate was determined non-invasively in mice using NIC kidney devices as described previously and in the supplemental methods.^30,31^

### Non-invasive and invasive assessment of cardiac function

All echocardiographic analyses were performed by Duke’s Cardiovascular Physiology Core using a Vevo 3100 imaging system (FUJIFILM VisualSonics, Toronto, Canada) please see supplemental methods for details.

### Serum chemistry

At study end, blood was collected from mice at the time of killing via cardiac puncture, transferred into Microvette heparin plasma tubes (Sarstedt, Germany) and centrifuged at 21,000g for 10 minutes. Plasma supernatants were collected and stored at −80 °C. Blood urea nitrogen (BUN), phosphate and hemoglobin were measured at the University of North Carolina Animal Histopathology and Lab Medicine Core as done previously with an Alfa Wassermann Vet Axcel^®^ Chemistry Analyzer (Alfa Wassermann Diagnostic Technologies, LLC, NJ, USA). Intact and C-terminal FGF23 levels were determined by ELISA (Immutopics, CA, USA) according to the manufacturers protocol. PTH levels were measured with the mouse PTH 1–84 ELISA kit (Immutopics, CA, USA) according to the manufactures protocol.

### Mitochondrial respiration

Mitochondrial isolation from frozen mouse hearts was performed as previously described and in the supplemental methods.^32^ High-resolution oxygen consumption rate (*J*O_2_) was assessed via the Oroboros Oxygraph-2K (Oroboros Instruments, Innsbruck, Austria) as previously described,^32^ with minor adjustments, see supplemental methods for details.

### RNA sequencing

RNA sequencing was performed in collaboration with Duke’s Center for Genomic and Computational Biology Core Facility. In brief, RNA-seq data was processed using the TrimGalore toolkit^33^ which employs Cutadapt^34^ to trim low-quality bases and Illumina sequencing adapters from the 3’ end of the reads. Only reads that were 20nt or longer after trimming were kept for further analysis. Reads were mapped to the GRCm38v73 version of the mouse genome and transcriptome^35^ using the STAR RNA-seq alignment tool^36^. Please see supplemental methods for further details.

### Proteomics

Proteomics of isolated cardiac mitochondria was performed in collaboration with Duke’s Proteomics and Metabolomics Core Facility. Please see supplemental methods for detailed description and references.

### Metabolomics

Metabolomic measurements were performed at the Metabolomics Core Laboratory at Duke Molecular Physiology Institute. Please see supplemental methods for detailed description and references.

### Transmission electron microscopy

Transmission electron microscopy was performed by Duke’s Center for Electron Microscopy. Please see supplemental methods for detailed description.

### Study approval

All animal protocols and experimental procedures for adenine diet in mice, and primary cardiomyocyte isolations from neonatal Sprague Dawley rats were approved by the Institutional Animal Care and Use Committees (IACUC) at Duke University. All animals were maintained in a ventilated rodent-housing system with temperature-controlled environments (22–23°C) with a 12-hour light/dark cycle and allowed ad libitum access to food and water. All protocols adhered to the Guide for Care and Use of Laboratory Animals to minimize pain and suffering. No animals were excluded from analysis.

### Statistical Analysis

All data are presented as means ± SEM. Identification of possible statistical outliers was performed by ROUT method (Q=1%) in GraphPad Prism. P < 0.05 was considered statistically significant. All data were analyzed using GraphPad Prism9 (Graphpad Software, CA, USA) followed by student’s T-tests or when appropriate by 2-way ANOVA.

## Results

### CKD alters cardiac mitochondrial structure prior to onset of LVH

**Figure 1:**
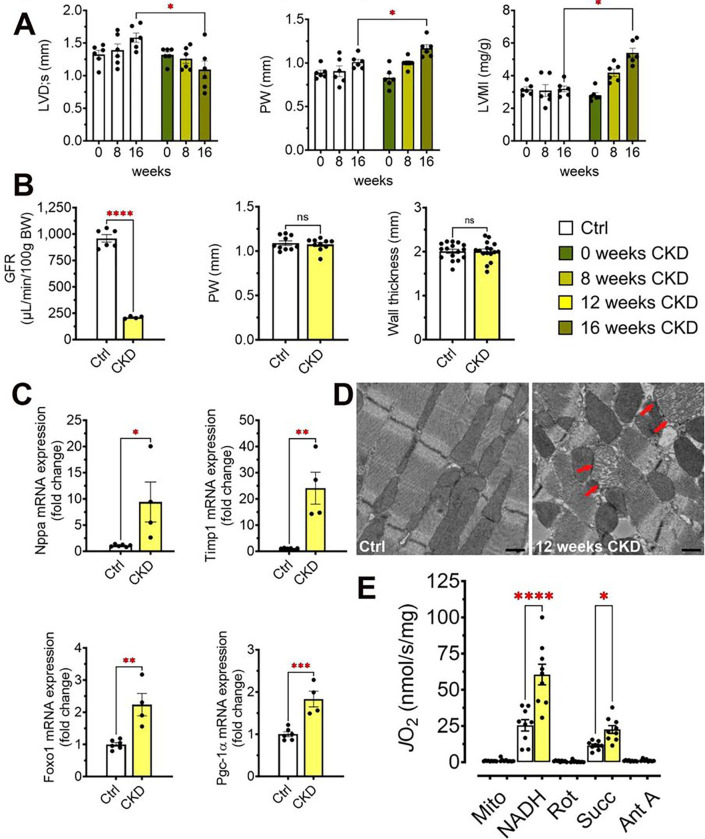
Cardiac function, remodeling and changes to cardiac mitochondria in CKD. Cardiac function of control and adenine fed mice was evaluated at the outset of the experiment at 8 weeks and at study termination at 16 weeks. Significant LVH was detectable at the final timepoint after 16 weeks CKD as indicated by left ventricular mass index (LVMI), posterior wall thickness (PW) and left ventricular end-systolic diameter (LVD;s) (A). After 12 weeks adenine diet, significant decrease in glomerular filtration rate (GFR) indicated kidney damage but no clear manifestation of structural LVH as indicated by the unchanged posterior wall thickness and overall wall thickness after 12 weeks on the adenine diet (B). Measurement of remodeling parameters *Nppa, Timp1, Foxo1* and *Pgc-1α* indicate that hypertrophic and fibrotic remodeling had been initiated at 12 weeks CKD (C). Electron microscopy revealed clear changes in mitochondrial morphology with misalignment of mitochondria and changes in cristae appearance (D). Mitochondrial respiration after 12 weeks of adenine diet indicated that respiration through complex I and II was significantly increased before structural cardiac remodeling was detectable (E). Bar graphs represent mean with SEM and individual values included in the graph. * indicate p< 0.05. Scale bar in D is 500nm.

To investigate cardiac metabolic remodeling in heart failure due to CKD, we analyzed cardiac structure and function in wild-type mice fed 0.2% adenine diet to induce CKD.^29^ Adenine-induced CKD caused progressive LVH over 16 weeks as indicated by increased LV mass index (LVMI), increased posterior and intra-septal wall thickness, and reduced systolic LV diameter (Figure 1A); LV function, marked by fractional shortening, was unchanged (Supplemental Figure S1).

After 12 weeks of consuming the adenine diet when glomerular filtration rate (GFR) was significantly reduced but no cardiac structural remodeling could be detected (Figure 1B), cardiac expression of *Nppa* and *Timp1* mRNA were increased, indicating that hypertrophic and fibrotic remodeling processes had already begun (Figure 1C). Interestingly, expression of the transcription factors PGC-1α and FOXO1, which are key regulators of myocardial metabolism and cardiac mitochondrial function, were also significantly increased in CKD versus controls (Figure 1C). At that time point, electron microscopy revealed grossly normal myofibrillar structures in the CKD myocardium, but mitochondria were misaligned, swollen and the cristae were disorganized, suggesting possible mitochondrial dysfunction (Figure 1D).

To characterize myocardial mitochondrial function, we assessed respiratory capacity across the electron transport system complexes. Respiration by both respiratory chain complex I (NADH supported) and complex II (succinate supported) was significantly increased in CKD hearts versus controls, suggesting that changes in cardiac mitochondrial function precede hypertrophic and fibrotic structural remodeling (Figure 1E). While many studies of heart failure describe reduced cardiac oxidative phosphorylation capacity, increases in mitochondrial respiration have also been reported in a rat model of right ventricular heart failure induced by pulmonary hypertension.^37^

In a second genetic model of progressive CKD that also develops LVH,^38^ we confirmed the presence of pathologic mitochondria and increased expression of profibrotic and hypertrophic markers in the hearts of C57BL6J/Col4a3^−/−^ mice with severe CKD (Supplemental Figure S2). These data support adverse effects of CKD on mitochondria across different models.

### CKD changes the cardiac mitoproteome and metabolome

**Figure 2:**
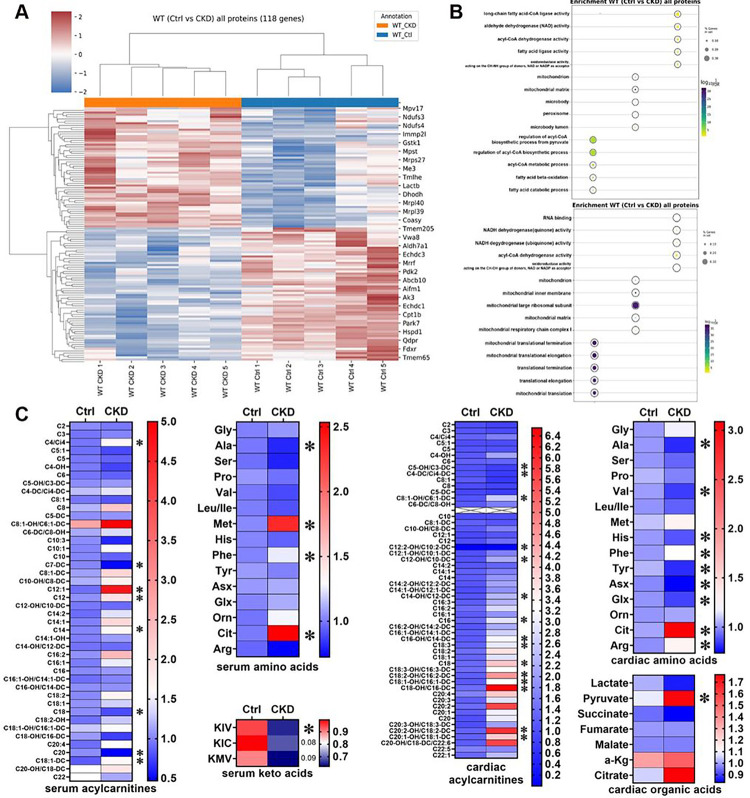
Changes to mitochondrial proteome and cardiac metabolome in CKD. CKD leads to significant changes in the mitochondrial proteome of wildtype after 12 weeks of adenine diet, before structural remodeling becomes detectable (A,B). A total of 118 mitochondrial genes were significantly regulated in the mitoproteome of mice with CKD compared to controls (A). KEGG pathway analysis showed that, downregulated proteins represented fatty acid metabolism, and amino acid degradation (B, top). The up-regulated genes were enriched in ribosomal processes and translation (B, bottom). Analysis of metabolomics also showed significant changes in serum acylcarnitines, amino acids and keto acids (C, left). Among upregulated serum amino acids were phenylalanine and citrulline. These were also upregulated in cardiac tissue (C, right). Here, more amino acids were significantly downregulated, leucine and isoleucine showed a downward trend (p = 0.07). In contrast to serum, more cardiac acylcarnitines were significantly downregulated. For organic acids from cardiac tissue of CKD mice only pyruvate reached significance but lactate and citrate showed trends (p= 0.07).

To investigate cardiac metabolic remodeling in response to CKD, we isolated mitochondria from the hearts of 12-week adenine fed CKD mice and assessed the cardiac mitoproteome using tandem mass spectrometry (Figure 2A). Using a previously reported mitochondrial enrichment factor,^39^ we achieved mitochondrial enrichment of approximately 75% (data not shown). Of the 781 mitochondrial genes identified across CKD and control mice, 56 were upregulated and 62 were downregulated in CKD hearts when compared to controls without CKD (Figure 2A). The downregulated proteins were significantly enriched in six different KEGG (Kyoto Encyclopedia of Genes and Genomes) pathways, fatty acid oxidation, acetyl-CoA metabolism, regulation of biosynthetic processes from pyruvate and NADPH, and anti-oxidant activity (Figure 2B, top). Upregulated proteins were involved in mitochondrial ribosomes and translation (Figure 2B, bottom). These results suggest that CKD induces functional changes in cardiac mitochondria.

To assess the cardiac metabolome in CKD, we performed targeted liquid chromatography-mass spectrometry (LC-MS) analysis of serum and heart tissue (Figure 2C). Several medium and long chain acylcarnitines (MLAC) were significantly altered in CKD (Figure 2C). In serum, MLAC were mostly upregulated whereas in cardiac tissue some MLACs were up and downregulated in when compared to controls. Several amino acids including arginine, phenylalanine and citrulline were increased in hearts and serum of CKD mice, whereas cardiac concentrations of branch chained amino acids including valine, leucine/isoleucine tended to be lower. In line, serum levels of branch chained keto acids were also significantly reduced in CKD. Significant changes were detected in cardiac organic acids and TCA intermediates. Pyruvate was significantly higher whereas lactate trended lower in CKD versus controls (Figure 2C). TCA cycle intermediates, including citrate, also trended to higher levels in CKD hearts (Figure 2C). Taken together, these data suggest that CKD alters fatty acid, amino acid and glucose metabolism in in the heart.

### FGF23-FGFR4 induce hypertrophic growth of bio-engineered cardio-bundles

**Figure 3:**
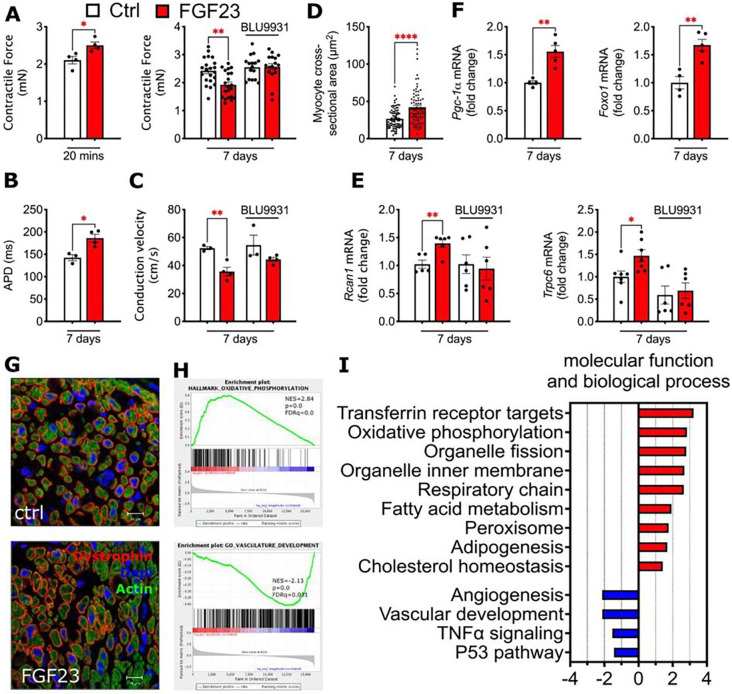
FGFR4 regulates metabolic transcription and hypertrophy in bio-engineered cardio bundles. Treatment of neonatal rat ventricular myocyte cardio-bundles with FGF23 for 20 minutes significantly increased contractile force, while 7 days of chronic treatment led to a significant reduction in contractile force that could be rescued by co-application of BLU9931, a selective FGFR4 inhibitor (A). Electrophysiological function was evaluated by pacing of cardio-bundles and application of Di-4-ANEPPS as voltage sensitive dye. Chronic exposure of cardio-bundles to FGF23 lead to significantly longer action potential durations (B). FGF23-treated bundles exhibited significantly lower conduction velocity that was normalized after co-application of BLU9931 (C). Beside functional changes, chronic FGF23 treatment also led to cardio-bundle hypertrophy indicated by the significant rise in cross-section (D,G) and increased expression of hypertrophic mRNA markers *Rcan1* and *Trpc6* (E). Increased expression of *Rcan1* and *Trpc6* was blocked by parallel treatment with BLU9931. Metabolic transcription factors that were increased in CKD mice, also increased in cardio-bundles after FGF23 treatment (F). Representative images of cardio-bundles indicate cellular hypertrophy after FGF23 treatment by increased myocyte cross-sections (G). Gene set enrichment analysis of control and FGF23 treated cardio-bundles showed an enrichment of metabolic pathways, particularly fatty acid metabolism, adipogenesis and cholesterol homeostasis (H). Additional enrichment was detected in pathways related to mitochondrial function, such as oxidative phosphorylation, respiratory chain, organelle fission and organelle inner membrane (I). Downregulated pathways after FGF23 treatment include angiogenesis, vascular development TNFα signaling and P53. Bar graphs represent mean with SEM and individual values included in the graph. * indicate p<0.05. Scale bars in G are 10 μm.

To investigate whether elevated FGF23-FGFR4 signaling might directly contribute to the cardiac metabolic remodeling observed in CKD, we studied cardio-bundles, 3-dimensional multicellular cylindrical tissues bio-engineered from neonatal rat ventricular myocytes (NRVM) and fibroblasts. The cardio-bundles spontaneously contract and exhibit mature functional properties similar to postnatal rat myocardium.^22^ Acute treatment of cardio-bundles with FGF23 (20 minutes) significantly increased contractility compared to vehicle, as reported previously (Figure 3A).^23^ In contrast, chronic FGF23 treatment (7 days) significantly decreased contractility; this effect was blocked by co-treatment with BLU9931, a small molecule isoform-specific inhibitor of FGFR4 (Figure 3A), confirming the specific involvement of the FGF23-FGFR4 axis.

To assess electrophysiological function, cardio-bundles were paced with a voltage-sensitive dye, followed by optical mapping of the action potential propagation. Chronic FGF23 treatment prolonged action potential duration (APD) compared to controls (Figure 3B). Conduction velocity was ~32% slower in FGF23-treated versus vehicle-treated cardio-bundles, an effect that was attenuated by BLU9931 (Figure 3C). The selected dose of BLU9931 had no effects on contractile force or conduction velocity of control cardio-bundles (Figure 3A,C), ensuring specificity for FGF23 induced, FGFR4-mediated effects.

Seven days of FGF23 treatment stimulated hypertrophic growth of cardio-bundles as indicated by increased cross-sectional area of individual myocytes and elevated mRNA expression of the hypertrophic markers TRPC6 and RCAN1 (Figure 3D,E,G); co-treatment with BLU9931 blocked these effects. Similar to the results from CKD mice, mRNA expression of metabolic transcription factors PGC-1α and FOXO1 increased in FGF23-treated cardio-bundles (Figure 3F).

To investigate the mechanism of FGF23-FGFR4 induced cardiac remodeling, we profiled changes in gene expression of cardio-bundles subjected to 7 days of FGF23 treatment using RNA sequencing. Metabolic pathways were highly enriched upon FGF23 treatment, including fatty acid metabolism, adipogenesis and cholesterol homeostasis (Figure 3H). In addition, FGF23-treated cardio-bundles showed strong enrichment in molecular processes related to mitochondrial structure and function including oxidative phosphorylation, respiratory chain, organelle fission and organelle inner membrane (Figure 3I). Downregulated genes involved molecular processes related to angiogenesis, vascular development, TNFα signaling, and the P53 pathway (Figure 3I). Taken together, these results suggest that FGF23 can induce *in vitro* changes in cardiac tissue remodeling that parallel those observed in mice with CKD and that these effects are mediated by FGFR4.

### FGF23-FGFR4 alters mitochondrial function in cultured cardiomyocytes

**Figure 4:**
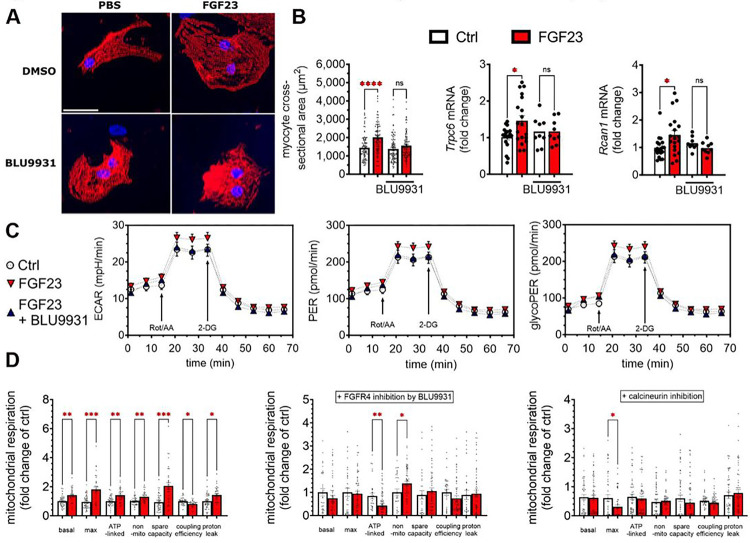
FGFR4 mediates metabolic remodeling in cultured cardiomyocytes Cultured neonatal rat ventricular myocytes (NRVM) esponded to 48h of FGF23 treatment with significant hypertrophy indicated by increased cross-sectional area and expression of pro-hypertrophic markers (A and B). Pro-hypertrophic mRNA expression and cellular hypertrophy could be mitigated by parallel treatment with the FGFR4-specific inhibitor BLU9931. Cardiac mitochondria isolated from NRVM treated with FGF23 for 1h, before observable hypertrophy takes place, were analyzed in a Seahorse XF analyzer for extracellular acidification rate (ECAR), elevated total proton efflux rates (PER) and glycolysis specific proton efflux rates (glycoPER) (C). ECAR was significantly higher in mitochondria from FGF23-treated cells, which could be reduced to control levels by BLU9931. PER showed elevated basal and compensatory glycolysis in mitochondria with FGF23 treatment; glycolysis-specific proton efflux was also increased. These FGF23 mediated effects were blocked by BLU9931 application. Seahorse mitochondrial stress test assay showed increased basal and maximal mitochondrial respiration after FGF23 treatment of NRVM (D). ATP production-linked, spare respiratory capacity and non-mitochondrial oxygen consumption rate increased in parallel after FGF23 treatment. The significant decrease in coupling efficiency and the increased proton leak indicate uncoupling of substrate oxidation and ATP synthesis after 1h of FGF23 treatment. Application of BLU9931 or the calcineurin inhibitor, cyclosporin A, prevented the changes to mitochondrial function caused by FGF23. Bar graphs represent mean with SEM and individual values included in the graph. * indicate p<0.05. Graphs in C represent 3 independent experiments.

Next, we determined if FGF23-FGFR4 directly modulates substrate utilization and mitochondrial respiration in cultured cardiomyocytes. NRVM were treated with FGF23 with and without BLU9931 for 48 hours. FGF23 induced hypertrophy of NRVM as determined by significant increases in the area of immunolabeled cells and elevated mRNA expression of the hypertrophic markers, TRPC6 and RCAN1 (Figure 4A,B), as previously reported.^12^ Co-treatment with BLU9931 blocked hypertrophic growth of cardiomyocytes, while treatment with BLU9931 by itself had no effect on NRVM (Figure 4A,B).

Activation of glycolysis is involved in cell growth, including cardiac remodeling.^40^ To determine if glycolysis is directly stimulated by FGF23 or increases indirectly in response to cellular hypertrophy, we treated NRVM with FGF23 for 1 hour, before cellular hypertrophy was present (Supplemental Figure S3). Using the Seahorse XF analyzer, we evaluated the extracellular acidification rate (ECAR) in NRVM as an indirect measure of glycolysis (Figure 4C). ECAR was significantly higher in FGF23-treated than control cells; this effect was abolished by BLU9931 (Figure 4C), which had no effect on its own (data not shown). Next, we assessed the glycolytic rate of NRVM, which removes the contribution of mitochondrial CO2 to ECAR and allows more accurate measurement of glycolysis. FGF23 significantly increased basal and compensatory glycolysis, as determined by elevated total proton efflux rates (PER) and glycolysis-specific proton efflux rates, whereas BLU9931 blocked these effects (glycoPER; Figure 4C).

To directly examine mitochondrial function in response to FGF23, we applied the Seahorse mitochondrial stress test assay to cultured cardiomyocytes. FGF23 significantly increased basal and maximum mitochondrial respiration in NRVM (Figure 4D). Similarly, ATP production-linked spare respiratory capacity and non-mitochondrial oxygen consumption rate (OCR) was higher in FGF23-treated cells (Figure 4D). In contrast, FGF23 significantly decreased coupling efficiency, indicating uncoupling of substrate oxidation and ATP synthesis (Figure 4D). The observed reduction in coupling efficiency was attributable to significantly increased proton leak (Figure 4D), which is the predominant mechanism for incomplete coupling.^41^ Pharmacologic inhibition of FGFR4 with BLU9931 and inhibition of calcineurin using cyclosporin A prevented the effects of FGF23 on mitochondrial respiration, implicating the FGFR4–PLCγ–calcineurin axis in the metabolic effects of FGF23 on glycolysis (Figure 4D).

### Activation of FGFR4 causes cardiac metabolic remodeling independently of CKD

**Figure 5:**
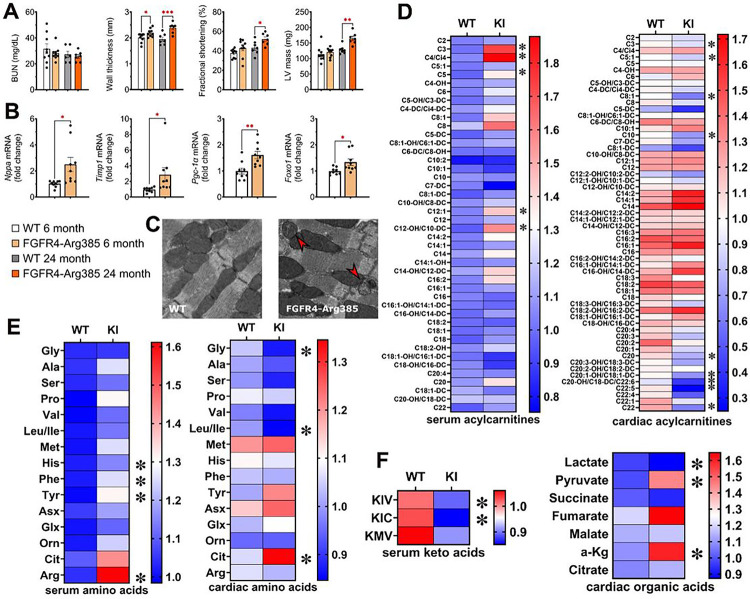
Cardiac function, remodeling and metabolomics of FGFR4-Arg385 mice in the absence of CKD. FGFR4-Arg385 mice did not have impaired kidney function as indicated by BUN values, but beginning LVH is detectable by increased wall thickness at 6 months of age. By 24 month of age, renal function remained unchanged and significant LVH/HFpEF was detected in FGFR4-Arg385 mice indicated by robust structural remodeling and increased fractional shortening (A). mRNA expression levels of remodeling, pro-fibrotic and pro-hypertrophic markers support initiation of cardiac remodeling at 6 months of age (B). Transmission electron microscopy showed similar changes in the mitochondria of six-month-old FGFR4-Arg385 mice as observed in mice with adenine induced CKD (C). Metabolomic analysis of FGFR4-Arg385 mice at 6 month of age showed significant increase in some serum acylcarnitines and reduction in cardiac MLACs (D). Similar to wildtype CKD animals, cardiac citrulline was upregulated while a number of other amino acids, including leucin and isoleucine, were downregulated (E). Reduction of serum keto acids was also in line with results obtained from the adenine CKD model. Organic acids also showed similar changes with a significant upregulation of pyruvate and a downregulation of lactate (F). Bar graphs represent mean with SEM and individual values included in the graph. * indicate p<0.05.

To test the hypothesis that FGFR4 activation contributes to cardiac metabolic remodeling, we investigated cardiac mitochondria in knock-in mice that express FGFR4-Arg385, which is a gain-of-function mutation of FGFR4.^12^ As we reported previously,^12^ there were no differences in kidney function or mineral metabolism between wild-type and FGFR4-Arg385 mice (Figure 5A, Supplemental Figure S4). Six-month-old FGFR4-Arg385 mice developed mild LVH, characterized by increased wall thickness and mRNA expression of hypertrophic and profibrotic markers, but overall LV mass, LV diameters and systolic and diastolic function were unchanged until 24 months of age when FGFR4-Arg385 mice developed overt LVH (Figure 5A,B, Supplemental Figure S4). Transmission electron microscopy revealed cardiac mitochondrial morphological changes in 6-month-old FGFR4-Arg385 mice that were similar to the changes observed in wild-type mice with CKD, including disorganized alignment and swollen mitochondrial cristae (Figure 5C). Analysis of the cardiac and serum metabolome in 6-month-old FGFR4-Arg385 mice demonstrated significant changes in organic acids, several MLAC, branch chained amino acids and branch chained keto acids in a similar pattern as observed in the CKD mice (Figure 5D–F). Taken together, these morphologic and metabolomic data suggest that expression of a constitutively active FGFR4 is sufficient to induce cardiac metabolic remodeling, which occurs prior to the onset of overt LVH and in a pattern that is similar to that observed in mice with CKD and wild-type FGFR4 expression.

### Global deletion of FGFR4 prevents remodeling of the cardiac mitoproteome in CKD

**Figure 6:**
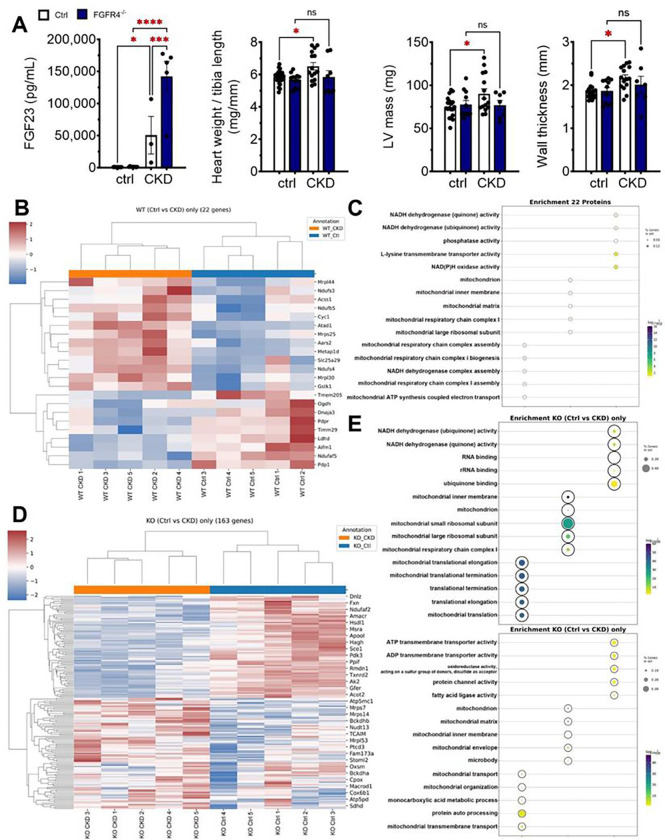
Global deletion of FGFR4 prevents LHV and changes to cardiac mitoproteome in CKD. Mice with global deletion of FGFR4 develop CKD to the same degree as control mice after 16 weeks of adenine diet as indicated by the rise in FGF23 (A). Wildtype animals developed LVH at 16 weeks with increased LV mass, wall thickness and ratio of heart weight/tibia length. These changes were absent in FGFR4−/− mice (B). Cardiac mitoproteome of wildtype and FGFR4−/− mice was evaluated after 12 weeks adenine feeding, before overt remodeling is observed. 22 proteins were significantly regulated in wildtype CKD mice, but were not changed in FGFR4−/− CKD mice, with 9 proteins downregulated and 13 upregulated (B). Analysis showed enrichment in pathways connected to mitochondrial respiration and function (C). Additionally, 163 proteins were identified that were only regulated in FGFR4−/− CKD mice with 83 downregulated and 57 upregulated proteins (D). Enrichment analysis showed a partial normalization of mitochondrial proteins in FGFR4−/− mice (E). Bar graphs represent mean with SEM and individual values included in the graph. * indicate p<0.05.

Global deletion of FGFR4 (FGFR4^−/−^) protects mice from LVH caused by chronic high phosphate diet.^23^ To test if FGFR4 deletion also protects against LVH in CKD, we subjected FGFR4^−/−^ mice to 16 weeks of adenine diet. As reported previously,^29^ all mice developed CKD with elevations in serum BUN and FGF23 levels (Figure 6A, Supplemental Figure S5). Control but not FGFR4^−/−^ mice developed pathological cardiac remodeling (Figure 6A) and elevated cardiac expression of pro-hypertrophic (*Nppb*) and pro-fibrotic (*Fn1*) markers (Supplemental Figure S5). Cardiac mRNA expression of metabolic transcription factor PGC-1α was upregulated in control but not FGFR4^−/−^ mice (Supplemental Figure S5).

We isolated mitochondria from the hearts of wild-type mice and FGFR4^−/−^ littermates with CKD and assessed the cardiac mitoproteome with LC-MS. The 9 proteins that were significantly downregulated and the 13 that were significantly upregulated in wild-type CKD mice were unchanged in FGFR4^−/−^ CKD mice, including proteins of mitochondrial respiration and function (Figure 6B,C). In addition, 83 proteins were downregulated and 57 were upregulated only in FGFR4^−/−^ CKD mice; enrichment analysis of these differentially expressed proteins suggests that deletion of FGFR4 upregulates pathways related to NADH activity, mitochondrial ribosomes, and mitochondrial translation and downregulates pathways linked to ATP transport, protein channel and fatty acid activity. (Figure 6D,E). Taken together, these results indicate that deletion of FGFR4 attenuates pathologic changes in cardiac mitochondrial composition in CKD.

### FGF23-FGFR4 signaling mediates cardiac metabolic remodeling in adenine-induced CKD

**Figure 7:**
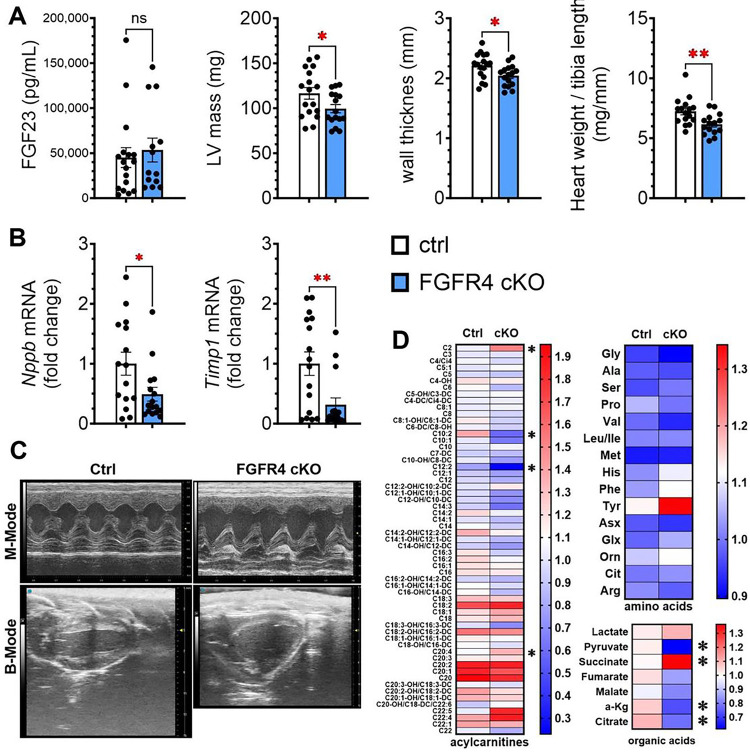
Cardiomyocyte expression of FGFR4 mediates metabolic remodeling in adenine-induced CKD. After 16 weeks of adenine diet, control animals and α-MHCMerCreMer-FGFR4flox mice developed kidney damage to a similar degree with elevated FGF23. LV mass, wall thickness and ratio of heart weight/tibia length were significantly lower in α-MHCMerCreMer-FGFR4flox mice than control animals (A). Cardiac-specific deletion of FGFR4 also significantly reduced the cardiac expression of pro-hypertrophic and pro-fibrotic markers (B). Echocardiography showed no structural remodeling or abnormalizes in the hearts of α-MHCMerCreMer-FGFR4flox, whereas hearts of control animals showed significant wall thickening and remodeling (C). Analysis of the cardiac metabolome showed elevation of a greater number of MLAC when compared to control animals. Expression levels of organic acids indicate a normalization of glucose utilization in α-MHCMerCreMer-FGFR4flox mice and reduction in cardiac pyruvate and citrate concentrations (D). Amino acids were unchanged between groups. Bar graphs represent mean with SEM and individual values included in the graph. * indicate p<0.05.

Mice with cardiomyocyte-specific deletion of FGFR4 do not develop LVH in response to repeated short-term (5 days) FGF23 injections.^13^ To determine if FGF23 and cardiac FGFR4 contribute to cardiac metabolic remodeling in CKD, we created mice with inducible cardiomyocyte-specific deletion of FGFR4 (α-MHC^MerCreMer^-FGFR4^flox^). α-MHC^MerCreMer^-FGFR4^flox^ mice were injected with tamoxifen and cardiac FGFR4 expression was evaluated 10 days later. FGFR4 mRNA expression was significantly reduced in the hearts but not the kidneys of α-MHC^MerCreMer^-FGFR4^flox^ mice compared to controls, indicating specific deletion of FGFR4 from cardiomyocytes (Supplemental Figure S6). After 16 weeks of adenine diet, all mice developed kidney injury to the same degree (Figure 7A, Supplemental Figure S6). Echocardiography revealed significantly lower LV mass, wall thickness and heart weight to tibia length ratio in α-MHC^MerCreMer^-FGFR4^flox^ mice versus controls (Figure 7A,C). In addition, cardiac mRNA expression of pro-hypertrophic (*Nppb*) and pro-fibrotic markers (*Timp1*) were significantly decreased in experimental mice versus controls (Figure 7B). Control but not α-MHC^MerCreMer^-FGFR4^flox^ mice developed diastolic dysfunction (Figure 7C, Supplemental Figure S6).

We analyzed the cardiac metabolome of α-MHC^MerCreMer^-FGFR4^flox^ mice and littermate controls (Figure 7D). Cardiomyocyte-specific deletion of FGFR4 elevated cardiac levels of MLAC. While amino acids and branch chained amino acids remained mostly unchanged, expression levels of organic acids suggested normalization of cardiac pyruvate, succinate and lactate concentrations. Taken together, these results indicate that blocking cardiac FGFR4 attenuates cardiac metabolic remodeling in CKD.

## Discussion

In this report, we demonstrate that cardiac mitochondrial dysfunction and metabolic remodeling are complications of CKD that precede structural remodeling of the heart that eventually culminates in LVH. Using bioengineered cardio-bundles, primary culture of NRVM, gain-of-function and loss-of function genetic mouse models, we further show that FGF23-mediated activation of FGFR4 is one potential underlying mechanism of cardiac mitochondrial dysfunction and metabolic remodeling in mice with CKD.

Studies that assessed cardiac metabolism in CKD are scarce and mostly addressed single metabolic pathways.^42–46^ In contrast, we used an agnostic approach to report on global changes in cardiac amino acids, acylcarnitines and organic acid metabolism in wild-type and FGFR4-deficient mice. Our results align with recent reports that compared cardiac metabolites from patients with heart failure with preserved ejection fraction (HFpEF) or reduced ejection fraction (HFrEF) and reports of different animal models.^47,48^ This suggests that HFpEF and cardiac metabolic remodeling with or without CKD likely share common mechanisms.

Genetic activation of FGFR4 caused HFpEF in the absence of kidney injury or elevated serum FGF23 levels in our study. Similar to CKD, metabolic changes manifested before overt LVH. Since the cardiac metabolome of CKD mice and FGFR4-Arg385 knock in mice were similar, we hypothesized that FGF23-FGFR4 signaling contributes to regulation of cardiac metabolism. This hypothesis is supported by our finding that LVH and HFpEF were attenuated and cardiac metabolism was normalized in mice with CKD overlaying global or cardiomyocyte-specific deletion of FGFR4 compared to mice with CKD and intact cardiac FGFR4. Our in vitro data in cardio-bundles and NRVM in which FGF23 drove contractile, electrical, and metabolic dysfunction that were prevented by a specific small molecule inhibitor of FGFR4 lend further support to our hypothesis.

Cardiac mitochondria play a major role in regulating cardiac energy homeostasis. Our analysis of cardiac mitochondria using transmission electron microscopy, proteomics and respirometry indicate that CKD causes substantial changes to cardiac mitochondrial structure and function before structural changes of the heart are evident. Interestingly, FGF23-mediated mitochondrial dysfunction is characterized by increased mitochondrial respiration in-vitro. These results are in line with our respirometry findings from CKD mice. Similar results have also been observed in right ventricular heart failure,^37^ in contrast to other heart failure models. Our results are supported by previous studies that described pathologic mitochondria in a rat model of CKD using high resolution imaging and increased respiration and proton leak in aged mitochondria.^49,50^ The pathological augmented basal respiration coupled with sustained proton leak likely augments bioenergetic stress, burdens the mitochondrial work load, ultimately resulting in a decline in respiratory efficiency. In addition, changes in mitochondrial oxidative catabolism have been described to drive adaptive changes in metabolic properties.^51^ Our results indicate that FGF23-mediated increases in mitochondrial respiration function as a retrograde signaling mechanism that ultimately increases glycolysis.

Limitations of this report include the lack of metabolic flux studies. Our static metabolomic results only provide a snapshot on cardiac metabolic pathways and do not allow a full interpretation of cardiac glycolysis and fatty acid metabolism in CKD. Moreover, we currently do not know how FGFR4 mediates its downstream metabolic effects. Enriched pathways in cardio-bundles treated with FGF23 included processes related to peroxisomal physiology and transferrin receptor biology, highlighting a possible mechanistic link between FGF23 and iron homeostasis, as has been reported previously.^52,53^ Increased respiration with elevated proton leak could indicate that increased production of reactive oxygen species plays a role in this mechanism. Additional experiments will be needed to elucidate the pathway from FGFR4 to mitochondrial dysfunction and whether it includes the PLCγ-calcineurin signaling cascade as indicated by the rise in TRPC6 and RCAN1 expression in cardio-bundles that we observed. Recently, renal glycolysis has been identified as a mammalian phosphate sensor and thus energy metabolism serves as a critical regulator of phosphate homeostasis that controls osseous FGF23 secretion via glycerol-3-phosphate.^54,55^ Our work now closes this “metabolic loop” by demonstrating that FGF23 itself exerts direct metabolic effects. HFpEF increases mortality in patients with CKD, but to date, we do not know enough about the early pathogenesis of HFpEF in CKD to support treat patients before cardiac structural changes become evident. Our findings that FGF23 mediates cardiac metabolic remodeling and mitochondrial dysfunction strongly supports the need to develop and test small molecule inhibitors of FGFR4 or its downstream effectors in preclinical models and early clinical studies for the treatment of cardiac metabolic remodeling to prevent subsequent development of LVH and HFpEF.

## Figures and Tables

**Table 1 :** Taq-man probes used for RNA quantification

Target	Assay ID
Eukaryotic 18S rRNA	4352930E
FGFR4	Mm01341851_g1
Fib	Mm01256744_m1
Foxol	Mm00490672_m1
Myh6	Mm00440359_m1
Myh7	Mm00600555_m1
Nppa	Mm01255747_g1
Pgc-1α	Mm01208835_m1
Rcanl	Rn00596606_m1
Timpl	Mm00441818_m1
Trpc6	Mm01176083_m1
β2-microglobulin	Mm00437762_m1

## Data Availability

All data presented in this manuscript will be made available to other researchers upon request to the corresponding author.

## References

[1] Coresh J. Prevalence of chronic kidney disease in the United States. JAMA 298, 2038–2047 (2007). 17986697 10.1001/jama.298.17.2038

[2] Hill N. R. Global Prevalence of Chronic Kidney Disease - A Systematic Review and Meta-Analysis. PLoS One 11, e0158765 (2016). 27383068 10.1371/journal.pone.0158765 PMC4934905

[3] Go A. S., Chertow G. M., Fan D., McCulloch C. E. & Hsu C. Chronic kidney disease and the risks of death, cardiovascular events, and hospitalization. N Engl J Med 351, 1296–1305 (2004). 15385656 10.1056/NEJMoa041031

[4] Weiner D. E. Chronic kidney disease as a risk factor for cardiovascular disease and all-cause mortality: a pooled analysis of community-based studies. J Am Soc Nephrol 15, 1307–1315 (2004). 15100371 10.1097/01.asn.0000123691.46138.e2

[5] Schefold J. C., Filippatos G., Hasenfuss G., Anker S. D. & von Haehling S. Heart failure and kidney dysfunction: epidemiology, mechanisms and management. Nat Rev Nephrol 12, 610–623 (2016). 27573728 10.1038/nrneph.2016.113

[6] Matsushita K. Epidemiology and risk of cardiovascular disease in populations with chronic kidney disease. Nat Rev Nephrol 18, 696–707 (2022). 36104509 10.1038/s41581-022-00616-6

[7] Erben R. G. Physiological Actions of Fibroblast Growth Factor-23. Front Endocrinol (Lausanne) 9, 267 (2018). 29892265 10.3389/fendo.2018.00267 PMC5985418

[8] Isakova T. Fibroblast growth factor 23 is elevated before parathyroid hormone and phosphate in chronic kidney disease. Kidney Int 79, 1370–1378 (2011). 21389978 10.1038/ki.2011.47 PMC3134393

[9] Faul C. FGF23 induces left ventricular hypertrophy. J Clin Invest 121, 4393–4408 (2011). 21985788 10.1172/JCI46122 PMC3204831

[10] Scialla J. J. Fibroblast growth factor-23 and cardiovascular events in CKD. J Am Soc Nephrol 25, 349–360 (2014). 24158986 10.1681/ASN.2013050465 PMC3904568

[11] Mehta R. Association of Fibroblast Growth Factor 23 With Atrial Fibrillation in Chronic Kidney Disease, From the Chronic Renal Insufficiency Cohort Study. JAMA Cardiol 1, 548–556 (2016). 27434583 10.1001/jamacardio.2016.1445 PMC4992989

[12] Grabner A. Activation of Cardiac Fibroblast Growth Factor Receptor 4 Causes Left Ventricular Hypertrophy. Cell Metabolism 22, 1020–1032 (2015). 26437603 10.1016/j.cmet.2015.09.002 PMC4670583

[13] Han X., Cai C., Xiao Z. & Quarles L. D. FGF23 induced left ventricular hypertrophy mediated by FGFR4 signaling in the myocardium is attenuated by soluble Klotho in mice. J Mol Cell Cardiol 138, 66–74 (2020). 31758962 10.1016/j.yjmcc.2019.11.149 PMC7195870

[14] Wilkins B. J. Calcineurin/NFAT Coupling Participates in Pathological, but not Physiological, Cardiac Hypertrophy. Circulation Research 94, 110–118 (2004). 14656927 10.1161/01.RES.0000109415.17511.18

[15] Akwo E. Association of Genetically Predicted Fibroblast Growth Factor-23 with Heart Failure: A Mendelian Randomization Study. Clin J Am Soc Nephrol 17, 1183–1193 (2022). 35902130 10.2215/CJN.00960122 PMC9435988

[16] Zhou B. & Tian R. Mitochondrial dysfunction in pathophysiology of heart failure. J Clin Invest 128, 3716–3726 (2018). 30124471 10.1172/JCI120849 PMC6118589

[17] Bertero E. & Maack C. Metabolic remodelling in heart failure. Nat Rev Cardiol 15, 457–470 (2018). 29915254 10.1038/s41569-018-0044-6

[18] McGarrah R. W. & White P. J. Branched-chain amino acids in cardiovascular disease. Nat Rev Cardiol 20, 77–89 (2023). 36064969 10.1038/s41569-022-00760-3 PMC10284296

[19] Nguyen T. D. & Schulze P. C. Cardiac Metabolism in Heart Failure and Implications for Uremic Cardiomyopathy. Circ Res 132, 1034–1049 (2023). 37053280 10.1161/CIRCRESAHA.123.321759

[20] Parker T. G., Packer S. E. & Schneider M. D. Peptide growth factors can provoke ‘fetal’ contractile protein gene expression in rat cardiac myocytes. https://www.jci.org/articles/view/114466/pdf (1990) doi:10.1172/JCI114466. PMC296452 1688886

[21] Helfer A. & Bursac N. Frame-hydrogel Methodology for Engineering Highly Functional Cardiac Tissue Constructs. Methods Mol Biol 2158, 171–186 (2021). 32857373 10.1007/978-1-0716-0668-1_13 PMC7672525

[22] Jackman C. P., Carlson A. L. & Bursac N. Dynamic culture yields engineered myocardium with near-adult functional output. Biomaterials 111, 66–79 (2016). 27723557 10.1016/j.biomaterials.2016.09.024 PMC5074846

[23] Grabner A. FGF23/FGFR4-mediated left ventricular hypertrophy is reversible. Sci Rep 7, 1993 (2017). 28512310 10.1038/s41598-017-02068-6 PMC5434018

[24] Weinstein M., Xu X., Ohyama K. & Deng C. X. FGFR-3 and FGFR-4 function cooperatively to direct alveogenesis in the murine lung. Development 125, 3615–3623 (1998). 9716527 10.1242/dev.125.18.3615

[25] Cosgrove D. Collagen COL4A3 knockout: a mouse model for autosomal Alport syndrome. Genes Dev 10, 2981–2992 (1996). 8956999 10.1101/gad.10.23.2981

[26] Seitzer N., Mayr T., Streit S. & Ullrich A. A Single Nucleotide Change in the Mouse Genome Accelerates Breast Cancer Progression. Cancer Research 70, 802–812 (2010). 20068154 10.1158/0008-5472.CAN-09-3239

[27] Sohal D. S. Temporally regulated and tissue-specific gene manipulations in the adult and embryonic heart using a tamoxifen-inducible Cre protein. Circ Res 89, 20–25 (2001). 11440973 10.1161/hh1301.092687

[28] Kawakami K. Persistent fibroblast growth factor 23 signalling in the parathyroid glands for secondary hyperparathyroidism in mice with chronic kidney disease. Sci Rep 7, 40534 (2017). 28094278 10.1038/srep40534 PMC5240111

[29] Taylor A. FGFR4 does not contribute to progression of chronic kidney disease. Sci Rep 9, 14023 (2019). 31575945 10.1038/s41598-019-50669-0 PMC6773883

[30] Ellery S. J., Cai X., Walker D. D., Dickinson H. & Kett M. M. Transcutaneous measurement of glomerular filtration rate in small rodents: Through the skin for the win? Nephrology 20, 117–123 (2015). 25388805 10.1111/nep.12363

[31] Rieg T. A High-throughput Method for Measurement of Glomerular Filtration Rate in Conscious Mice. J Vis Exp (2013) doi:10.3791/50330. PMC3679673 23712131

[32] McLaughlin K. L. Bioenergetic Phenotyping of DEN-Induced Hepatocellular Carcinoma Reveals a Link Between Adenylate Kinase Isoform Expression and Reduced Complex I-Supported Respiration. Front Oncol 12, 919880 (2022). 35756609 10.3389/fonc.2022.919880 PMC9213884

[33] Babraham Bioinformatics - Trim Galore! http://www.bioinformatics.babraham.ac.uk/projects/trim_galore/.

[34] Martin M. Cutadapt removes adapter sequences from high-throughput sequencing reads. EMBnet.journal 17, 10–12 (2011).

[35] Kersey P. J. Ensembl Genomes: an integrative resource for genome-scale data from non-vertebrate species. Nucleic Acids Research 40, D91–D97 (2012). 22067447 10.1093/nar/gkr895 PMC3245118

[36] Dobin A. STAR: ultrafast universal RNA-seq aligner. Bioinformatics 29, 15–21 (2013). 23104886 10.1093/bioinformatics/bts635 PMC3530905

[37] Redout E. M. Right-ventricular failure is associated with increased mitochondrial complex II activity and production of reactive oxygen species. Cardiovasc Res 75, 770–781 (2007). 17582388 10.1016/j.cardiores.2007.05.012

[38] Neuburg S. Genetic background influences cardiac phenotype in murine chronic kidney disease. Nephrology Dialysis Transplantation 33, 1129–1137 (2018). 10.1093/ndt/gfx332 PMC6030849 29309658

[39] McLaughlin K. L. Novel approach to quantify mitochondrial content and intrinsic bioenergetic efficiency across organs. Sci Rep 10, 17599 (2020). 33077793 10.1038/s41598-020-74718-1 PMC7572412

[40] Gibb A. A. & Hill B. G. Metabolic Coordination of Physiological and Pathological Cardiac Remodeling. Circulation Research 123, 107–128 (2018). 29929976 10.1161/CIRCRESAHA.118.312017 PMC6023588

[41] Brand M. D. Uncoupling to survive? The role of mitochondrial inefficiency in ageing. Experimental Gerontology 35, 811–820 (2000). 11053672 10.1016/s0531-5565(00)00135-2

[42] Nishimura M. Prediction of cardiac death in hemodialysis patients by myocardial fatty acid imaging. J Am Coll Cardiol 51, 139–145 (2008). 18191738 10.1016/j.jacc.2007.08.057

[43] Dilsizian V. & Fink J. C. Deleterious effect of altered myocardial fatty acid metabolism in kidney disease. J Am Coll Cardiol 51, 146–148 (2008). 18191739 10.1016/j.jacc.2007.09.032

[44] Reddy V., Bhandari S. & Seymour A.-M. L. Myocardial function, energy provision, and carnitine deficiency in experimental uremia. J Am Soc Nephrol 18, 84–92 (2007). 17182887 10.1681/ASN.2005080876

[45] Raine A. E., Seymour A. M., Roberts A. F., Radda G. K. & Ledingham J. G. Impairment of cardiac function and energetics in experimental renal failure. J Clin Invest 92, 2934–2940 (1993). 8254048 10.1172/JCI116916 PMC288497

[46] Smith K., Semple D., Aksentijevic D., Bhandari S. & Seymour A.-M. L. Functional and metabolic adaptation in uraemic cardiomyopathy. Front Biosci (Elite Ed) 2, 1492–1501 (2010). 20515820 10.2741/e208

[47] Hahn V. S. Myocardial Metabolomics of Human Heart Failure With Preserved Ejection Fraction. Circulation 147, 1147–1161 (2023). 36856044 10.1161/CIRCULATIONAHA.122.061846 PMC11059242

[48] Schiattarella G. G. Nitrosative Stress Drives Heart Failure with Preserved Ejection Fraction. Nature 568, 351–356 (2019). 30971818 10.1038/s41586-019-1100-z PMC6635957

[49] Zhang H. Reduction of elevated proton leak rejuvenates mitochondria in the aged cardiomyocyte. eLife 9, e60827 (2020). 33319746 10.7554/eLife.60827 PMC7738186

[50] Bigelman E. Pathological presentation of cardiac mitochondria in a rat model for chronic kidney disease. PLoS One 13, e0198196 (2018). 29889834 10.1371/journal.pone.0198196 PMC5995391

[51] Pereyra A. S. Skeletal muscle undergoes fiber type metabolic switch without myosin heavy chain switch in response to defective fatty acid oxidation. Mol Metab 59, 101456 (2022). 35150906 10.1016/j.molmet.2022.101456 PMC8898976

[52] Wolf M., Koch T. A. & Bregman D. B. Effects of iron deficiency anemia and its treatment on fibroblast growth factor 23 and phosphate homeostasis in women. J Bone Miner Res 28, 1793–1803 (2013). 23505057 10.1002/jbmr.1923

[53] Courbon G. Bone-derived C-terminal FGF23 cleaved peptides increase iron availability in acute inflammation. Blood 142, 106–118 (2023). 37053547 10.1182/blood.2022018475 PMC10356820

[54] Simic P. Glycerol-3-phosphate is an FGF23 regulator derived from the injured kidney. J Clin Invest 130, 1513–1526 (2020). 32065590 10.1172/JCI131190 PMC7269595

[55] Zhou W. Kidney glycolysis serves as a mammalian phosphate sensor that maintains phosphate homeostasis. J Clin Invest (2023) doi:10.1172/JCI164610. PMC10104895 36821389

